# Deregulation of the CDX2-KLF4 axis in acute myeloid leukemia and colon cancer

**DOI:** 10.18632/oncotarget.896

**Published:** 2013-02-21

**Authors:** Arefeh Rouhi, Stefan Fröhling

**Affiliations:** Department of Internal Medicine III, Ulm University, Ulm, Germany; Department of Translational Oncology, National Center for Tumor Diseases and German Cancer Research Center, Heidelberg, Germany

Acute myeloid leukemia (AML), the most common acute leukemia in adults, is characterized by enormous genetic and biological heterogeneity. As a consequence, the identification of unifying pathogenetic mechanisms and common targets for therapy has been next to impossible. We and others previously showed that the non-clustered homeobox gene *CDX2*, which encodes a transcription factor involved in embryonic development and intestinal cell homeostasis, is aberrantly expressed in nearly all cases of AML and promotes leukemogenesis, whereas CDX2 expression is absent in normal hematopoietic stem and progenitor cells (HSPC) [1,2]. These findings had pointed to the potential of targeting CDX2 in AML; however, little was known about the effectors through which CDX2 contributes to AML development, and specific strategies for interfering with CDX2 function in patients had remained elusive.

We recently addressed these unresolved issues [3]. Analysis of the transcriptional effects of Cdx2 in primary murine HSPC and a mouse model of Cdx2-driven leukemia, followed by intersection with data from genome-wide expression analysis of a large AML patient cohort identified the zinc-finger protein KLF4, an established tumor suppressor in certain epithelial and B-cell malignancies [4-6], as an essential CDX2 target. CDX2 silenced *KLF4* transcription through binding to the *KLF4* upstream regulatory region and a decrease in histone 3 lysine 4 trimethylation (H3K4me3) at the *KLF4* promoter mediated by the H3K4 demethylase KDM5B. In support of a tumor-suppressive role for KLF4 in CDX2-driven AML, exogenous KLF4 induced cell cycle arrest, apoptosis, and differentiation preferentially in cultured myeloid leukemia cells that express CDX2. Furthermore, KLF4 inhibited the development of Cdx2-induced murine leukemias in competitive and non-competitive bone marrow transplantation experiments. A chemical genomic analysis based on the Connectivity Map [7] revealed that the transcriptional changes induced by CDX2 in hematopoietic cells were counteracted by drugs that stimulate the nuclear receptor PPARγ. Of potential clinical-translational relevance, PPARγ agonists also upregulated KLF4 and were toxic to CDX2-expressing myeloid leukemia cells, which were found to display altered PPARγ signaling both in vitro and in vivo, but not to normal HSPC.

In addition to elucidating the role of CDX2 in leukemia, these findings also provided insight into the opposing effects of CDX2 in AML and colon cancer, where CDX2 can function as a tumor suppressor [8]. Specifically, we observed that in colonic epithelial cells, KLF4 is positively regulated by CDX2, and consistent with its tissue-specific properties, CDX2 was found to bind to distinct sites in the *KLF4* regulatory region in AML versus colon cancer cells, possibly due to different DNA methylation patterns and DNA accessibility, inducing antagonistic changes in the levels of H3K4me3 at the *KLF4* promoter.

In summary, these studies (i) delineate transcriptional programs associated with aberrant CDX2 expression in hematopoietic cells; (ii) uncover *KLF4* as a previously unrecognized myeloid leukemia suppressor gene that is silenced by CDX2; (iii) identify reactivation of KLF4, through modulation of PPARγ signaling, as a new therapeutic modality that could impact treatment in a large proportion of AML patients; and (iv) indicate that transcriptional regulators like CDX2 may have opposing effects on carcinogenesis in different tissues due to variations in the epigenetic landscape and differential regulation of their downstream targets.

Together with recent data demonstrating the leukemogenic activity of HLX, another homeodomain transcription factor overexpressed in the majority of AML cases [9], these findings raise the possibility that widespread deregulation of non-clustered homeobox genes may contribute to the molecular “environment” that HSPC need to acquire specific “driver” mutations and propagate leukemic growth. Alternatively, *CDX2* and *HLX*, and possibly other related genes, may be part of a common effector pathway that lies downstream of different primary leukemogenic events. Despite these insights, a number of questions remain. For example, it is still unclear how CDX2 is regulated in AML, supporting unbiased screens for the upstream events that initiate aberrant CDX2 expression using tools such as large-scale RNA interference. Second, it will be interesting to study in vivo how CDX2 overexpression alters normal hematopoietic development, i.e. to characterize the effects of CDX2 on the various HSPC compartments, differentiation and survival of HSPC and their susceptibility to leukemogenic mutations. Third, the antagonistic duality of CDX2 function in AML versus colon cancer warrants further study, in particular the potential role of cell type-specific posttranslational modifications of CDX2 or context-dependent coactivators/repressors that may share DNA binding sites with CDX2 and thereby enable differential regulation of target genes such as *KLF4*. Finally, it remains to be seen whether the link between aberrant CDX2 expression, deregulated PPARγ signaling, and sensitivity to PPARγ agonist treatment can be exploited to improve the outcome of patients with AML, an aggressive disease that is notoriously difficult to treat.
